# Tea polyphenols inhibit the growth and virulence properties of *Fusobacterium nucleatum*

**DOI:** 10.1038/srep44815

**Published:** 2017-03-21

**Authors:** Amel Ben Lagha, Bruno Haas, Daniel Grenier

**Affiliations:** 1 Oral Ecology Research Group, Faculty of Dentistry, Université Laval, Quebec City, QC, Canada

## Abstract

*Fusobacterium nucleatum* plays a key role in creating the pathogenic subgingival biofilm that initiates destructive periodontitis. It is also a common resident of the human gastrointestinal tract and has been associated with inflammatory bowel disease. The aim of the present study was to investigate the effects of green and black tea extracts as well as two of their bioactive components, EGCG and theaflavins, on the growth and virulence properties of *F. nucleatum*. The tea extracts and components displayed various degrees of antibacterial activity that may involve damage to the bacterial cell membrane and the chelation of iron. They also prevented biofilm formation by *F. nucleatum* at concentrations that did not interfere with bacterial growth. In addition, the treatment of a pre-formed *F. nucleatum* biofilm with the green tea extract and EGCG caused a time-dependent decrease in biofilm viability. The green and black tea extracts, EGCG, and theaflavins decreased the adherence of *F. nucleatum* to oral epithelial cells and matrix proteins. Moreover, these tea components also attenuated *F. nucleatum*-mediated hemolysis and hydrogen sulfide production, two other virulence factors expressed by this bacterium. In summary, this study showed that tea polyphenols may be of interest for treating *F. nucleatum*-associated disorders.

Periodontitis is a chronic inflammatory disease affecting the supporting tissues of the teeth, ultimately leading to tooth loss. A limited group of bacteria, mostly strictly anaerobic Gram-negative species, are the primary etiological agents of periodontitis^1^. More specifically, *Fusobacterium nucleatum* is a fusiform bacterium that increases in numbers in subgingival sites affected by chronic periodontitis^2^. It plays a key role in subgingival biofilm formation by bridging the early colonizers (streptococci and actinomyces) and the late colonizers (*Porphyromonas gingivalis, Tannerella forsythia, Treponema denticola*)^3^ that make up the red complex initially described by Socransky *et al*. and that are strongly associated with active periodontal lesions^4^. Given its central role in biofilm formation, *F. nucleatum* may represent a key target for controlling biofilm formation. The outer membrane of *F. nucleatum* contains a number of adhesins that are involved in adherence to salivary proteins, bacteria, and host cells^5^,^6^,^7^. Additional virulence properties associated with *F. nucleatum* include hemolytic activity^8^ and the ability to produce hydrogen sulfide (H_2_S)^9^. *F. nucleatum* is well known for its invasive properties^10^, which may allow it to enter the bloodstream, migrate, and cause infections elsewhere in the body^11^. *F. nucleatum* is also a common resident of the human gastrointestinal tract^12^. Over the past few years, several studies have shown that this bacterial species increases in numbers in patients affected with inflammatory bowel disease and may contribute to the pathogenesis of colorectal cancer^12^,^13^,^14^.

Periodontitis treatments are aimed at eliminating the periodontal bacterial load by supragingival and subgingival mechanical debridement. However, to treat some categories of patients such as smokers and diabetics and to efficiently remove periodontopathogens colonizing the deep periodontal pockets, adjunctive antibiotic treatments may be required^15^. The development of antibiotic resistance in periodontopathogens is a major issue given that these bacteria may migrate from the oral cavity to other organs where they may cause infections or transfer their antibiotic resistances to other bacterial species. *F. nucleatum* strains that are resistant to amoxicillin, clindamycin, and metronidazole have already been isolated^16^,^17^,^18^. Given the emergence of antibiotic-resistant bacteria, new, preferably inexpensive, alternatives to conventional antibiotics must be developed. Tea, the most-consumed beverage worldwide next to water, is brewed from the leaves of the *Camellia sinensis* plant^19^,^20^. While green tea (non-fermented with a high catechin content) is mainly consumed in China and Japan, black tea (fermented with a high theaflavin content) is more popular in Western countries^19^. Numerous human, animal, and *in vitro* studies have shown that tea polyphenols may help reduce the risk of cardiovascular diseases and some forms of cancers, and may also improve physiological functions by lowering blood pressure, increasing bone density, and controlling body weight^19^,^20^,^21^. Tea polyphenols have also shown great promise as antimicrobial agents that are active against both Gram-positive and Gram-negative pathogens^22^,^23^.

In a previous study, we showed that green and black tea extracts, epigallocatechin-3-gallate (EGCG), and theaflavins reduce *F. nucleatum*-induced NF-κB activation in monocytes^24^. In addition, pre-treating macrophages with tea polyphenols prior to incubating them with *F. nucleatum* significantly inhibited the secretion of pro-inflammatory cytokines and matrix metalloproteinases^24^. Given these findings indicating that tea polyphenols can attenuate the *F. nucleatum*-induced host inflammatory response, the aim of the present study was to investigate their effects on the growth and virulence properties of *F. nucleatum*.

## Results

The antibacterial activities of green and black tea extracts, EGCG, and theaflavins against *F. nucleatum* were determined using a broth microdilution assay (Table 1). The MIC and MBC values of the green tea extract were 1000 μg/ml while those of the black tea extract were 2000 μg/ml. EGCG and the theaflavins had a MIC of 500 μg/ml. The bactericidal activity of the theaflavins was slightly higher than that of EGCG, with MBCs of 500 μg/ml and 1000 μg/ml, respectively.

Calcein-AM, a fluorescent dye, was used to investigate the effects of tea polyphenols on the membrane integrity of *F. nucleatum*. After a 1-h incubation, with the exception of the black tea extract, the addition of the other tea fractions to calcein-AM-loaded bacterial cells caused a marked dose-dependent release of fluorescence (Table 2). The largest release of calcein-AM was observed with EGCG and, to a lesser extent, the green tea extract. When the incubation was extended to 3 h, the black tea extract induced the release of calcein-AM, although much less than the other tea fractions.

**Figure 1:**
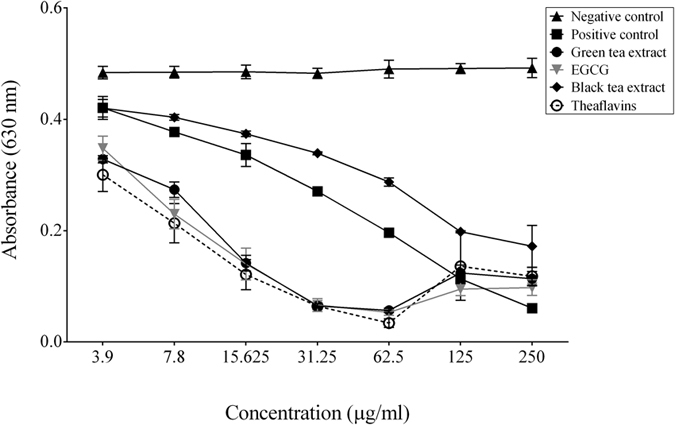
Iron-chelating activity of the green tea extract, black tea extract, EGCG, and theaflavins determined using a siderophore colorimetric assay. A decrease in A_630_ occurs when a strong chelator removes the iron from the chrome azurol sulfate dye. Ferrichrome, a siderophore produced by *U. sphaerogena,* was used as positive control. Results are expressed as the means ± SD of triplicate assays from two independent experiments. All values are significantly different from the negative control (p < 0.01).

We then determined whether tea polyphenols possess the ability to chelate iron, an essential nutrient for most bacteria^25^, which would be an additional antibacterial mechanism. All the tea compounds dose-dependently chelated iron in a universal siderophore assay (Fig. 1). The iron-chelating activities of the green tea extract, EGCG, and theaflavins were more pronounced than that of ferrichrome, a reference siderophore produced by *Ustilago sphaerogena.*

**Figure 2:**
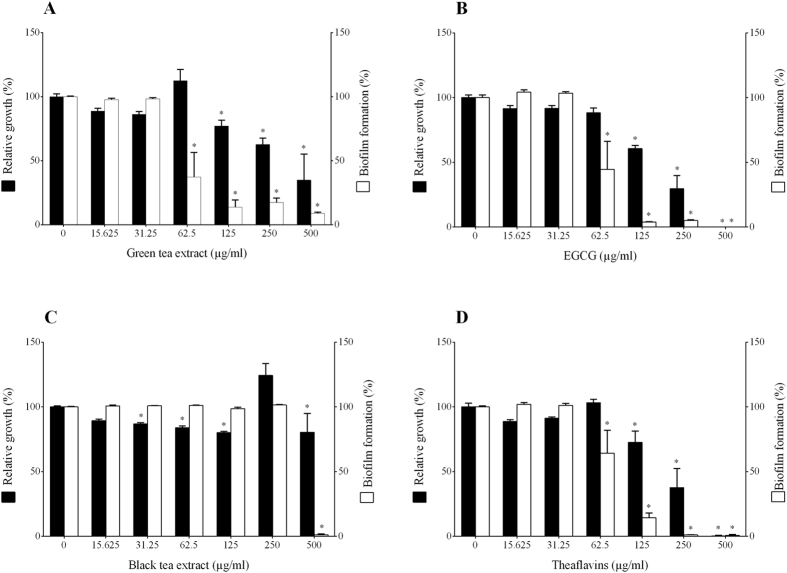
Effects of the green tea extract, black tea extract, EGCG, and theaflavins on the growth of and biofilm formation by *F. nucleatum*. A value of 100% was assigned to growth and biofilm formation obtained with *F. nucleatum* in the absence of tea polyphenols. Results are expressed as the means ± SD of triplicate assays from three independent experiments. *Significantly different from the control (p < 0.01).

The inhibition of *F. nucleatum* biofilm formation by tea polyphenols was evaluated by crystal violet staining following growth in a 96-well microplate. The green tea extract, EGCG and the theaflavins were the most effective at preventing biofilm formation, while the black tea extract was the least effective, with inhibition only being observed at the highest concentration tested (500 μg/ml) (Fig. 2). When added at a concentration of 62.5 μg/ml, the green tea extract and EGCG reduced biofilm formation by 62.5% and 55.4%, respectively, while bacterial growth was not significantly affected. At this concentration, the black tea extract had no effect on biofilm formation, while the theaflavins reduced biofilm formation by 35.8%.

We then tested the capacity of the tea polyphenols to reduce the viability of a *F. nucleatum* biofilm. Pre-formed 48-h biofilms were exposed for different times (5, 15, and 30 min) to tea polyphenols at their MBC. Viability was then assessed using a luminescence assay measuring ATP, an indicator of metabolically active bacteria. The green tea extract and EGCG caused a time-dependent decrease in biofilm viability (Table 3). A 15-min treatment with the green tea extract and EGCG killed 36.3% and 42.7% of the biofilm-embedded bacteria, respectively. The effect was more important than that obtained with chlorhexidine, which was used as a control. The biofilm had to be treated for 30 min with the black tea extract and theaflavins to induce a significant reduction in viability. Crystal violet staining showed that none of the treatments eradicated the biofilm (data not shown).

**Figure 3:**
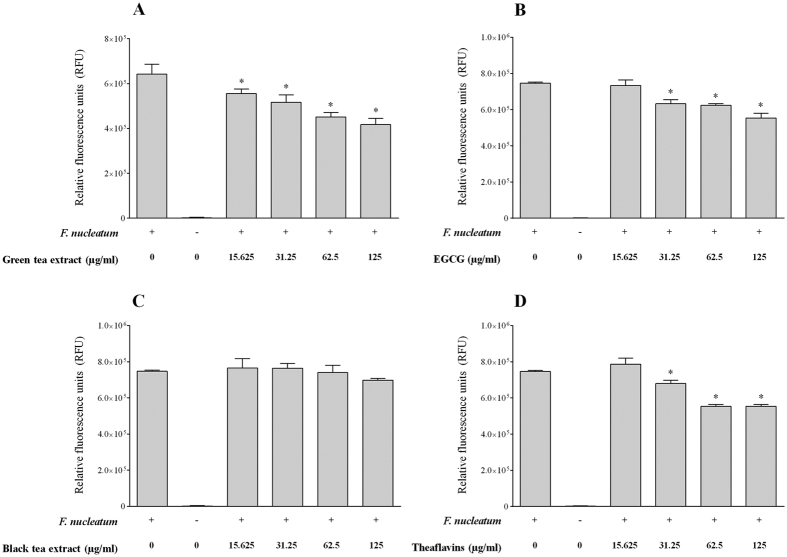
Effects of the green tea extract, black tea extract, EGCG, and theaflavins on the adherence of *F. nucleatum* to oral epithelial cells. Results are expressed as the means ± SD of triplicate assays from two independent experiments. *Significant decrease (p < 0.01) compared with the untreated control.

**Figure 4:**
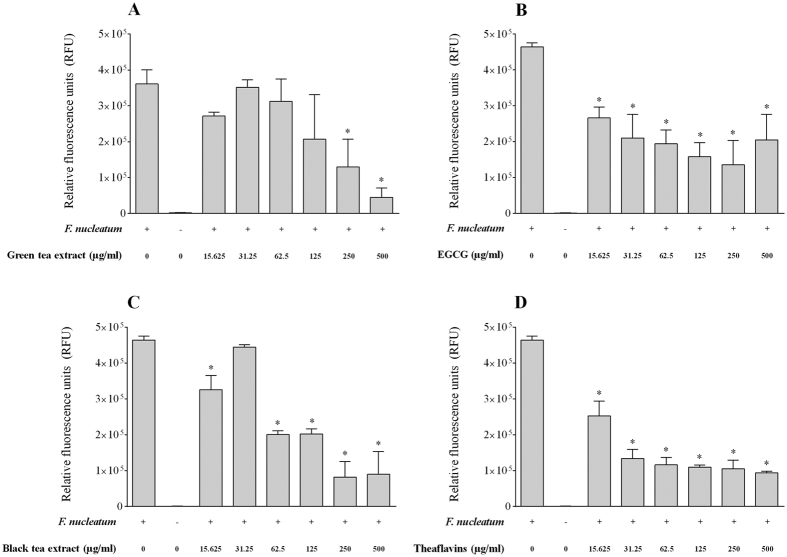
Effects of the green tea extract, black tea extract, EGCG, and theaflavins on the adherence of *F. nucleatum* to extracellular matrix proteins (Matrigel^®^). Results are expressed as the means ± SD of triplicate assays from two independent experiments. *Significant decrease (p < 0.01) compared with the untreated control.

The effect of tea polyphenols on the adherence of *F. nucleatum* to a confluent monolayer of oral epithelial cells was investigated using fluorescein-labeled bacterial cells. We first determined non-cytotoxic concentrations of tea polyphenols to be used with the GMSM-K cell line. As reported in Table 4, no decrease in cell viability was observed for oral epithelial cells treated (24 h) with either the green tea extract, the black tea extract, EGCG or the theaflavins at concentrations ≤ 125 μg/ml. Interestingly, an increased proliferation of cells was associated with a treatment with the green tea extract or EGCG at the lowest concentration (31.25 μg/ml) (Table 4). The adherence of *F. nucleatum* to oral epithelial cells was dose-dependently inhibited in the presence of the tea compounds (Fig. 3). At a concentration of 125 μg/ml, the green tea extract, the black tea extract, EGCG, and the theaflavins inhibited adherence by 34.9%, 6.5%, 25.8%, and 25.9%, respectively. We then evaluated whether tea polyphenols could also inhibit the adherence of *F. nucleatum* to a polystyrene surface coated with Matrigel^®^, a basement membrane matrix model. At a concentration of 250 μg/ml, the green tea extract, the black tea extract, EGCG, and the theaflavins inhibited adherence to the Matrigel^®^-coated polystyrene surface by 64.0%, 82.4%, 70.8%, and 77.3%, respectively (Fig. 4).

**Figure 5:**
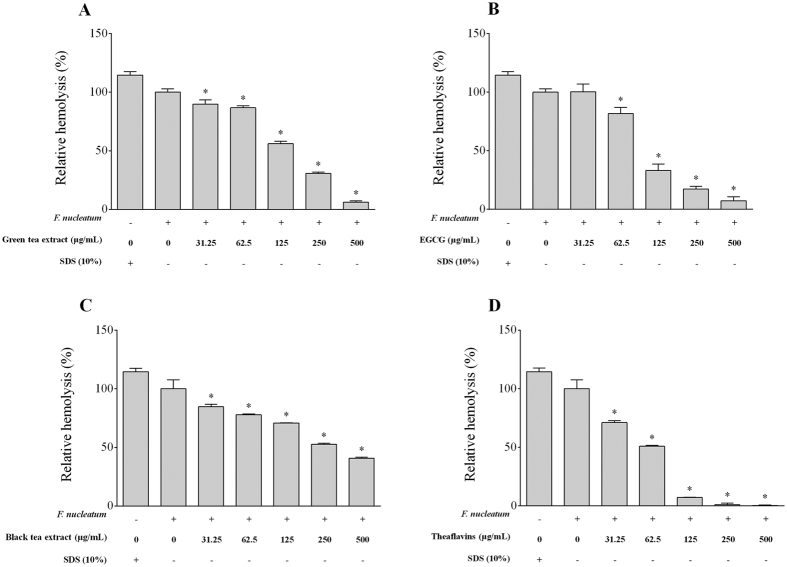
Effects of the green tea extract, black tea extract, EGCG, and theaflavins on the hemolytic activity of *F. nucleatum.* SDS (10%) was used as positive control to induce complete lysis of sheep erythrocytes. A value of 100% was assigned to the hemolysis by *F. nucleatum* in the absence of tea polyphenols. Results are expressed as the means ± SD of triplicate assays from three independent experiments. *Significantly different from the control (p < 0.01).

In a hemolytic assay using sheep red blood cells, *F. nucleatum* caused hemolysis similar to that obtained with SDS (positive control) (Fig. 5). All the compounds tested inhibited hemolysis to various degrees, with the theaflavins being the most potent. At a concentration of 125 μg/ml, the theaflavins, the green tea extract, the black tea extract, and EGCG inhibited hemolysis by 92.9%, 43.9%, 29.2%, and 67.0%, respectively.

**Figure 6:**
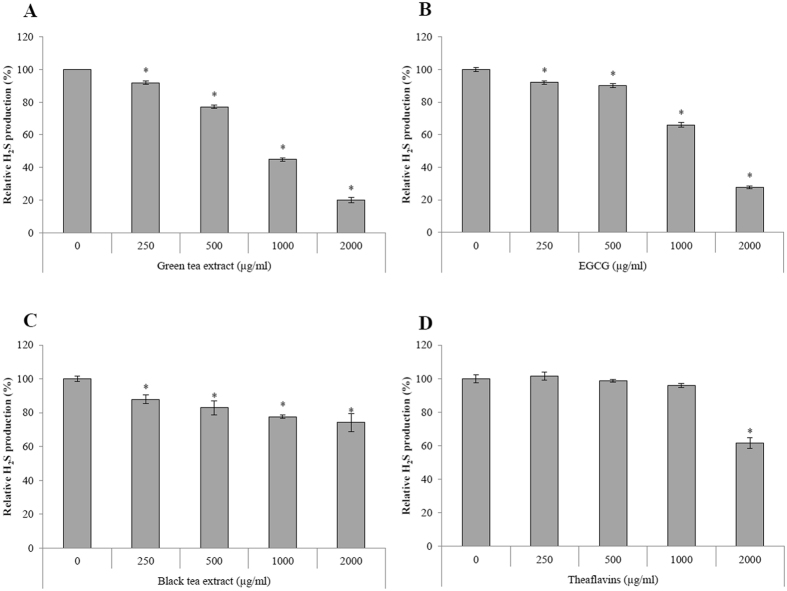
Effects of the green tea extract, black tea extract, EGCG, and theaflavins on H_2_S production by *F. nucleatum.* A value of 100% was assigned to H_2_S production by *F. nucleatum* in the absence of tea polyphenols. Results are expressed as the means ± SD of triplicate assays from three independent experiments. *Significantly different from the control (p < 0.01).

Lastly, we investigated the effect of tea polyphenols on H_2_S production by *F. nucleatum* using a colorimetric assay with cysteine and homocysteine as substrates. The green tea extract and EGCG were the most effective, reducing H_2_S production by 80.1% and 72.5%, respectively, at a concentration of 2000 μg/ml (Fig. 6). They also caused weak but significant inhibition at concentrations ≤500 μg/ml. The black tea extract and theaflavins were much less effective in inhibiting H_2_S production.

## Discussion

*F. nucleatum* plays a central role in creating the pathogenic subgingival biofilm that induces chronic inflammation that, in turn, leads to destructive periodontitis^3^. *F. nucleatum* can also be isolated from extra-oral infections and has been associated with inflammatory bowel disease and colorectal cancer^11^,^12^,^13^,^14^. In a previous study^24^, we used a macrophage model to show that tea polyphenols, including EGCG and theaflavins, can attenuate the inflammatory response induced by *F. nucleatum*. To further investigate the potential of tea polyphenols to treat *F. nucleatum*-associated disorders, the present study was designed to evaluate the effects of green and black tea extracts as well as two of their bioactive components, EGCG and theaflavins, on the growth and virulence properties of *F. nucleatum*.

We first showed that the growth of *F. nucleatum* is inhibited by tea polyphenols, more efficiently by EGCG and theaflavins, with a MIC of 500 μg/ml. Interestingly, we recently reported that EGCG and theaflavins may exert antibacterial activity indirectly by inducing the secretion of human β-defensins (−1, −2, −3) by oral keratinocytes^26^,^27^. These antimicrobial peptides are active against periodontopathogenic bacteria, including *F. nucleatum*^28^. We then provided evidence that tea polyphenols may exert antibacterial effects against *F. nucleatum* in two ways. First, we used calcein-AM-loaded *F. nucleatum* cells to show that tea polyphenols appear to damage the integrity of the bacterial cell membrane and cause cell lysis. Second, we showed that tea polyphenols possess the capacity to chelate iron, an essential nutrient, which may contribute to their antibacterial activity. It cannot be excluded that additional mechanisms may also contribute to the antibacterial activity of tea polyphenols. For instance, Navarro-Martinez *et al*.^29^ provided evidence that the antibacterial action of EGCG may be related to its ability to inhibit cytoplasmic dihydrofolate reductase, an enzyme involved in the synthesis of nucleic acid precursors. All of these results suggest that the antimicrobial property of tea polyphenols is likely multifactorial. EGCG and theaflavins have been reported to inhibit the growth of additional periodontopathogens, including *P. gingivalis, Prevotella intermedia,* and *Aggregatibacter actinomycetemcomitans*^26^,^30^. Interestingly, we recently showed that EGCG and theaflavins can potentiate the effects of conventional antibiotics (metronidazole, tetracycline) used in periodontal therapy^26^,^30^.

The ability of *F. nucleatum* to coaggregate and form biofilms plays a key role in the development of subgingival plaque associated with periodontitis. Moreover, when *F. nucleatum* is embedded in a biofilm, it is more resistance to both mechanical removal and antimicrobial agents than planktonic cells. Given that biofilm formation by *F. nucleatum* is a critical step in periodontal disease development, compounds that prevent biofilm formation or kill biofilm-embedded bacteria can be used to prevent periodontitis. The results of the present study showed that, at concentrations below their MIC values, the green tea extract, EGCG, theaflavins and, to a lesser extent, the black tea extract, inhibit biofilm formation by *F. nucleatum*. Moreover, exposure of a pre-formed *F. nucleatum* biofilm to MBCs of the green tea extract and EGCG resulted in a time-dependent decrease in biofilm viability. This effect was much less pronounced with the green tea extract and theaflavins. Similar observations with respect to the effect of EGCG on biofilm formation and the killing of bacteria embedded in biofilms have also been reported for *P. gingivalis*, another periodontopathogenic bacterial species^31^. All these results indicate that tea polyphenols show promise as an anti-biofilm agent.

The adherence of *F. nucleatum* to the oral mucosa is the initial step in the invasion of tissues that may result in extra-oral infections^32^. The present study showed that tea polyphenols can decrease the adherence of *F. nucleatum* to oral epithelial cells. Although the exact mechanism of inhibition was not investigated, it may result from the binding of tea polyphenols to the surface of bacteria and/or epithelial cells, hiding receptors or causing modifications to cell surface charges. A quantitative RT-PCR analysis of *F. nucleatum* cells exposed to tea polyphenols revealed no effect on the expression of genes (*fap2, fadA, aid1*) encoding adhesins known to be involved in host colonization (data not shown). The location of the basement membrane matrix between the sulcular epithelium and the subjacent connective tissues makes it the last potential barrier to bacterial translocation from the pocket into the connective tissues^33^. The ability of *F. nucleatum* to adhere to several extracellular matrix proteins, including laminin and type IV collagen, has been previously reported^33^. In this study, we showed that all tea polyphenols tested can attenuate the adherence of *F. nucleatum* to Matrigel^®^, a well-known basement membrane model.

The ability of *F. nucleatum* to lyse erythrocytes and release hemoglobin is considered a virulence determinant since it provides an iron source to *F. nucleatum* and periodontopathogens that promotes their proliferation in periodontal pockets^34^. Interestingly, the tea polyphenols dose-dependently inhibited the hemolytic activity of *F. nucleatum*. In a previous study, Grinberg *et al*. reported that tea polyphenols from black and green teas have a protective effect on red blood cells challenged with exogenous oxidants that induce lysis^35^. It was suggested that tea polyphenols are capable of forming a redox-inactive complex with iron, accounting for the antioxidant activity of tea polyphenols.

*F. nucleatum* is involved in halitosis (oral malodor) through its ability to produce volatile sulfur compounds such as H_2_S, which may also contribute to the pathogenesis of periodontitis because it is highly toxic for immune and mucosal cells and enhances their capacity to induce inflammatory reactions^36^,^37^,^38^. Natural substances with the capacity to control bacterial H_2_S production may thus attenuate halitosis and the development and severity of periodontal disease. The green tea extract and EGCG markedly reduced H_2_S production by *F. nucleatum*. The black tea extract and theaflavins also inhibited H_2_S production, albeit to a lesser extent. Interestingly, two previous clinical studies reported that the use of green tea mouthwashes reduce H_2_S production and consequently halitosis^39^,^40^.

In summary, the present study showed that green and black tea extracts, EGCG, and theaflavins possess antibacterial and anti-adherence activities against *F. nucleatum.* More specifically, they reduced adherence to gingival epithelial cells and biofilm formation, and killed biofilm-embedded *F. nucleatum* cells. Tea polyphenols also attenuated the hemolytic activity of and H_2_S production by *F. nucleatum*. Our results suggested that green and black tea polyphenols may be promising adjuncts for the treatment of periodontitis.

## Materials and Methods

### Tea polyphenols

According to the manufacturer, the commercial green and black tea extracts (Hangzhou Gosun Biotechnologies Co., China) had a polyphenol content of 98.4% and 92%, respectively. Stock solutions were freshly prepared by dissolving the tea powder (20 mg of green tea extract or 10 mg of black tea extract) in 1 ml of warm sterile distilled water and were filtered through 0.22-μm pore size membrane filters. EGCG (Sigma-Aldrich, Canada), the predominant catechin in the green tea extract, was also dissolved in sterile distilled water at a concentration of 10 mg/ml and was sterilized by filtration. The theaflavin preparation (DeHe Biotechnology Co., China) is a mixture of theaflavin, theaflavin-3-gallate, theaflavin-3′-gallate, and theaflavin-3,3′-digallate with more than 80% purity according to the product datasheet. A stock solution was prepared by dissolving 20 mg of powder in 1 ml of 95% ethanol.

### Bacteria and growth conditions

*F. nucleatum* ATCC 25586 was grown anaerobically (80% N_2_, 10% CO_2_, 10% H_2_) at 37 °C in Todd-Hewitt broth (THB; Becton Dickinson, Canada) supplemented with 0.001% hemin and 0.0001% vitamin K.

### Determination of the minimal inhibitory and minimal bactericidal concentrations

The minimal inhibitory concentration (MIC) and minimal bactericidal concentration (MBC) values of the green and black tea extracts, EGCG, and theaflavins against *F. nucleatum* were determined using a microplate dilution assay as described previously^41^.

### Cell membrane permeability assay

The effects of the green and black tea extracts (500 μg/ml), EGCG (250 μg/ml), and theaflavins (250 μg/ml) on the integrity of the cell membrane of *F. nucleatum* were determined using the intracellular dye calcein acetoxymethyl ester (calcein-AM) (Sigma-Aldrich, Canada) as previously described^42^.

### Determination of siderophore activity

The universal siderophore assay of Schwyn and Neils^43^ was used to measure the iron-chelating activity of the green and black tea extracts, EGCG, and theaflavins (250 μg/ml to 3.9 μg/ml). Ferrichrome (Sigma-Aldrich), a siderophore produced by *U. sphaerogena*, was used as a positive control^44^.

### Biofilm formation, viability, and eradication

A 24-h culture of *F. nucleatum* was diluted in fresh broth medium to obtain an optical density at 655 nm (OD_655_) of 0.2. Samples (100 μl) were added to the wells of a 96-well tissue culture plate (Sarstedt, USA) containing 100 μl aliquots of two-fold serial dilutions (500 to 3.9 μg/ml) of green tea extract, black tea extract, EGCG, or theaflavins in culture medium. Control wells with no compounds were also inoculated. After a 48-h incubation at 37 °C under anaerobic conditions, spent medium and free-floating bacteria were removed by aspiration using a 26 g needle, and the wells were washed three times with distilled water. The *F. nucleatum* biofilms were stained with 0.4% crystal violet (100 μl) for 15 min. The wells were washed four times with distilled water to remove unbound crystal violet dye and were dried for 2 h at 37 °C. After adding 100 μl of 95% (v/v) ethanol to each well, the plate was shaken for 10 min to release the stain from the biofilms and the absorbance at 550 nm (A_550_) was recorded. The capacities of the compounds to kill *F. nucleatum* cells embedded in the biofilm and to eradicate the biofilms were also investigated. Briefly, 48-h *F. nucleatum* biofilms were produced as described above and were treated for 5, 15, or 30 min with the compounds at final concentrations corresponding to their MBC values. One series of biofilms was then stained with crystal violet as described above to assess the eradication of the biofilms. A second series of biofilms was used to assess the viability of the *F. nucleatum* cells using a commercial luminescence assay (BacTiter Glo™ Microbial Cell Viability Assay; Promega, USA) according to the manufacturer’s protocol.

### Adherence to oral epithelial cells and a basement membrane matrix model

To determine the effect of tea polyphenols on the adherence of *F. nucleatum* to human oral epithelial cells, the bacterial cells were first labeled with fluorescein isothyocyanate (FITC) as described in a previous study^45^. FITC-labeled bacteria were suspended in Dulbecco’s modified Eagle’s medium (DMEM) supplemented with 10% heat-inactivated fetal bovine serum (FBS). The immortalized human oral epithelial cell line GMSM-K, which has been previously characterized by Gilchrist *et al*.^46^, was cultured in DMEM supplemented with 10% heat-inactivated FBS and 100 μg/ml of penicillin G-streptomycin, and incubated at 37 °C in an atmosphere of 5% CO_2_. Non-cytotoxic concentrations of tea polyphenols were first determined using an MTT (3-[4,5-diethylthiazol-2-yl]-2,5diphenyltetrazolium bromide) colorimetric assay (Roche Diagnostics, Canada). Then, epithelial cells (1.5 × 10^6^ cells/ml) were seeded and cultured in 96-well clear bottom black microplates. After an overnight incubation, the medium was removed by aspiration, and the confluent cell monolayers were pre-incubated with two-fold serial dilutions of the tea compounds (125 μg/ml to 15.625 in DMEM) for 30 min at 37 °C in a 5% CO_2_ atmosphere prior to adding FITC-labeled *F. nucleatum* cells at a multiplicity of infection (MOI) of 10^3^. The microplates were then incubated for a further 4 h at 37 °C in a 5% CO_2_ atmosphere. Unbound bacteria were removed by aspiration, and the wells were washed twice with PBS. Relative fluorescence units (RUF; excitation wavelength 495 nm; emission wavelength 525 nm) corresponding to the level of bacterial adherence were determined using a Synergy 2 microplate reader (BioTek Instruments, USA). Wells with no *F. nucleatum* were used as controls to measure basal autofluorescence. Control wells without tea polyphenols were used to determine 100% adherence values. The effect of tea polyphenols on the adherence of *F. nucleatum* to Matrigel™ (BD Biosciences, USA), a basement membrane matrix model composed of several extracellular matrix proteins, including laminin, type IV collagen, heparin sulfate proteoglycans, and entactin, was also evaluated. One hundred μl of Matrigel™ diluted 1/10 in ice-cold PBS was added to the wells of 96-well clear bottom black microplates (Greiner Bio-One North America, USA). After gelling (room temperature for 2 h), the Matrigel™ was washed twice with PBS, and two-fold serial dilutions of the tea compounds (250 to 15.625 μg/ml in PBS) were pipetted on top of the gel. After 30 min, 100 μl of FITC-labeled *F. nucleatum* cells (OD_660_ = 0.5) were added to the wells, and the plate was incubated for a further 4 h at 37 °C in a 5% CO_2_ atmosphere. Unbound bacteria were removed by aspiration, and the wells were washed twice with PBS. Bacterial adherence was monitored as described above.

### Hemolytic activity

Fresh sheep erythrocytes (Nutra-Bact, Canada) were harvested from whole blood by centrifugation (600 × *g* for 5 min), washed three times in PBS, and suspended in PBS to a concentration of 2% (v/v). Equal volumes (1 ml) of erythrocytes, bacterial cells (OD_660_ = 1.0 in PBS), and two-fold serial dilutions of tea polyphenols (500 to 31.25 μg/ml) were mixed together and were incubated at 37 °C for 4 h. PBS replaced the bacteria in the negative control, while 10% sodium dodecyl sulfate (SDS) instead of bacteria was used for the positive control (complete lysis of erythrocytes). Following the incubation at 37 °C, the mixtures were incubated at 4 °C for 1 h. They were then centrifuged (10,000 × *g* for 5 min), and the absorbances of the supernatants were measured at 540 nm (A_540_).

### Hydrogen sulfide (H_2_S) production

The production of H_2_S from cysteine and homocysteine by *F. nucleatum* was evaluated using a previously described protocol, with modifications^9^. Cells from a 24-h culture of *F. nucleatum* were harvested by centrifugation and were resuspended in oxygen-free 0.1% Tryptone to an OD_660_ = 1.0. Aliquots (50 μl) of the bacterial suspension were pipetted into the wells of a 96-well microplate. The green and black tea extracts, EGCG, and theaflavins were added (50 μl) to the wells along with a reaction mixture containing 400 mM triethanolamine-HCl, 20 μM pyridoxal-5′-phosphate, 10 mM bismuth III chloride, 10 mM L-cysteine, 10 mM DL-homocysteine, and 20 mM EDTA (pH 8). The plate was sealed with a plastic sheet and was incubated under anaerobic conditions for 1 h at 37 °C. The final concentrations of the tea compounds ranged from 2,000 to 250 μg/ml. H_2_S production was quantified by measuring the absorbance at 620 nm (A_620_) using a BioTek Synergy 2 microplate reader. The control values (without bacteria) were subtracted from the corresponding reactions to compensate for background absorbance by the medium.

### Statistical analysis

Unless indicated otherwise, all experiments were performed in triplicate and the means ± standard deviations (SD) were calculated. Three independent experiments were performed, and representative sets of data are presented. Statistical analyses were performed using a one-way analysis of variance with a post hoc Bonferroni multiple comparison test (GraphPad Software Inc., USA). All results were considered statistically significant at *p* < 0.01.

## Additional Information

**How to cite this article:** Ben Lagha, A. *et al*. Tea polyphenols inhibit the growth and virulence properties of *Fusobacterium nucleatum. Sci. Rep.* **7**, 44815; doi: 10.1038/srep44815 (2017).

**Publisher's note:** Springer Nature remains neutral with regard to jurisdictional claims in published maps and institutional affiliations.

## Figures and Tables

**Table 1:** MIC and MBC values of green tea extract, EGCG, black tea extract, and theaflavins against *F. nucleatum*.

Compounds	MIC (μg/ml)	MBC (μg/ml)
Green tea extract	1000	1000
EGCG	500	1000
Black tea extract	2000	2000
Theaflavins	500	500

**Table 2:** Effects of green tea extract, EGCG, black tea extract, and theaflavins on the release of calcein-AM from *F. nucleatum* cells.

Compound	Calcein-AM release	
	1 h	3 h
Green tea extract (μg/ml)		
250	297,616 ± 4,952	879,478 ± 18,081
125	243,369 ± 6,469	888,497 ± 28,797
62.5	170,951 ± 2,812	785,010 ± 20,653
31.25	101,531 ± 2,337	590,671 ± 18,437
EGCG (μg/ml)		
250	322,657 ± 15,202	888,821 ± 34,773
125	268,855 ± 10,228	908,765 ± 33,533
62.5	198,635 ± 8,584	853,987 ± 51,929
31.25	122,593 ± 4,639	655,047 ± 22,622
Black tea extract (μg/ml)		
250	641 ± 693	114,663 ± 3,118
125	786 ± 1,055	63,281 ± 5,986
62.5	0 ± 0	36,223 ± 1,106
31.25	0 ± 0	21,596 ± 2,864
Theaflavins (μg/ml)		
250	72,752 ± 3,844	897,622 ± 40,271
125	48,122 ± 1,923	731,390 ± 10,143
62.5	26,403 ± 1,092	494,629 ± 21,680
31.25	11,270 ± 1,518	299,153 ± 6,660

**Table 3:** Effects of green tea extract, EGCG, black tea extract, and theaflavins on killing of a *F. nucleatum* pre-formed biofilm.

Compound	MBC (μg/ml)	Relative killing (%)		
		5 min	15 min	30 min
Green tea extract	1000	24.25 ± 11. 18	36.33 ± 14.45	54.40 ± 9.13
EGCG	1000	18.04 ± 15.11	42.70 ± 2.34	62.44 ± 7.03
Black tea extract	2000	0 ± 24.39	0 ± 7.43	34.6 ± 9.15
Theaflavins	500	0 ± 13.55	0 ± 16.48	41.26 ± 11.93
Chlorhexidine	4	0 ± 8.60	8.35 ± 2.63	14.22 ± 1.50

Chlorhexidine was used as a positive control.

**Table 4:** Effects of green tea extract, EGCG, black tea extract, and theaflavins on viability of oral epithelial cells (GMSM-K cell line) as determined with a MTT assay.

Compounds	Cell viability (%)
None	100 ± 23.12
125	95.78 ± 3.2
62.5	117.0 ± 10.9
31.25	144.2 ± 15.04
EGCG (μg/ml)	
125	87.54 ± 8.0
62.5	132.6 ± 8.4
31.25	177.1 ± 4.7
Black tea extract (μg/ml)	
125	85.87 ± 1.9
62.5	90.22 ± 6.3
31.25	102.6 ± 5.5
Theaflavins (μg/ml)	
125	83.9 ± 9.65
62.5	92.65 ± 5.3
31.25	112.7 ± 7.0
